# Effect and Safety of Interferon for Hepatocellular Carcinoma: A Systematic Review and Meta-Analysis

**DOI:** 10.1371/journal.pone.0061361

**Published:** 2013-09-17

**Authors:** Liping Zhuang, Xiantao Zeng, Zongguo Yang, Zhiqiang Meng

**Affiliations:** 1 Department of Integrative Medicine, Fudan University Shanghai Cancer Center, Department of Oncology, Shanghai Medical College, Fudan University, Shanghai, China; 2 Department of Stomatology, Taihe Hospital, Hubei University of Medicne, Shiyan, Hubei Province, China; 3 Department of Traditional Chinese Medicine, Shanghai Public Health Clinical Center Affiliated to Fudan University, Shanghai, China; Icahn School of Medicine at Mount Sinai, United States of America

## Abstract

**Background:**

The effect of interferon(IFN) in the management of hepatocellular carcinoma (HCC) remains controversial, and no clear recommendations have been proposed.

**Objectives:**

To evaluate the effect and safety of IFN for HCC.

**Methods:**

PubMed, OvidSP, and Cochrane Library were searched from their establishment date until August 30, 2012. Studies that met the inclusion criteria were systematically evaluated and then subjected to meta-analysis.

**Results:**

Thirteen randomized control trials (RCTs) involving 1344 patients were eligible for this study. When IFN was used as an adjuvant therapy for HCC patients after curative therapy, the meta-analysis showed that IFN reduced the 1-, 2-, 3-, 4-, and 5-year recurrence rates. Subgroup analysis showed that IFN reduced the 2-, 3-, 4-, and 5-year recurrence rates of hepatitis C viral (HCV)-related HCC. The effect of IFN for on hepatitis B virus(HBV)-related HCC patients could not be determined because of isufficient data. After surgical resection, adjuvant IFN therapy reduced the 4- and 5- recurrence rates. All studies reported that IFN could not improve the overall survival of HCV-realated HCC patients after curative therapies. Only one study showed that IFN was associated with better overall survival in HCC patients after curative therapy and subgroup of HCC patients after surgical resection. Thus, meta-analysis was not performed. Different treatment options were used as control to study the effect of IFN for intermediate and advanced HCC patients, thus meta-analysis was not appropriate. All included studies, except for one, reported that IFN treatment was well tolerated.

**Conclusions:**

After curative therapies, adjuvant IFN reduced the recurrence of HCC. IFN did not improve the survival of HCV-related HCC patients after curative therapy. Whether IFN is effective for intermediate and advanced HCC patients could not be determined because of insufficient data. The toxicity of IFN was acceptable.

## Introduction

Hepatocellular carcinoma (HCC), the most common type of hepatobiliary cancer, is highly lethal. The global incidence of HCC has continuously increased, with Asian countries accounting for almost 80% of victims worldwide [1]–[3]. Strong geographic correlations have been found in the risk factors for HCC development. For example, chronic hepatitis B viral(HBV) infection is the leading cause of HCC in Asia and Africa, whereas hepatitis C viral(HCV) infection and alcoholic cirrhosis are the leading causues of HCC in developed countries [4].

The optimal therapy for HCC remains surgical resection, with a 5-year survival rate of 40% to 70% [5]–[6] and a 5-year recurrence rate of 75% to100% [7]–8. However, surgical resection is only available for less than 20% of patients [9]. Nonresectable HCC patients can benefit from multimodality treatment options [10]–[11]. However, an appropriate multimodality treatment remains unavailable to date. The presence of underlying liver disease is also an important issue. After an invasive primary therapy, reactivation of viral replication would induce further deterioration of hepatic function and may result in poor treatment tolerance and poor outcome [12]–[13]. Viral load is a useful prognostic marker for HBV-related HCC, and a low level of viral load represents a favorable outcome. In their gene-expression signature study, Hoshida et al. [14] suggested that the extent of liver damage and the presence of protein-inflammatory milieu may lead to an increased risk for disease recurrence in postoperative HCC patients. Therefore, antiviral treatment might be a beneficial choice.

The biological properties of interferon (IFN), including antiviral, anti-tumor, and immunomodulatory activities, have been studied extensively [15]. Three distinct classes of IFN subtypes are recognized: type I (IFN-α and IFN-β), type II (IFN-γ), and type III (IFN-λ). These types are distinguished by their differing receptors. However, only type I is widely used in therapy. The most common use of IFN type I is in the treatment of chronic viral hepatitis. IFN type I can improve the underlying liver pathology and reduce the risk of HCC in patients with HBV or HCV infection [16]–[19]. IFN type I improvs the outcomes of melanoma and renal cell carcinoma [20]. However, the effect of IFN in the management of HCC is still controversial, and no clear recommendations have been proposed.

In this study, we performed a systematic review and meta-analysis of randomized control trials (RCTs) to confirm the potential effect of IFN therapy on HCC patients. This study was reported following the PRISMA (Preferred Reporting Items for Systematic Reviews and Meta-Analyses) statement [21].

## Methods

### Literature search

PubMed, OvidSP, and Cochrane Library were searched up to August 30, 2012 for relevant citations. The MESH terms used in these databases were “carcinoma, hepatocellular” and “interferons”. Article type restriction of RCT was conducted. Two authors independently performed the literature search.

### Study selection

Two authors independently selected trials and discussed with each other when inconsistencies were found. Studies were selected based on the following inclusion criteria: (i) study design, RCT; (ii) participants, HCC patients; (iii) interventions, whether IFN was used was the mere treatment difference between the two groups regardless of the combined therapy; (iv) outcomes, to study the effect of IFN on HCC patients after curative therapy, the primary and secondary outcome measures were recurrence and survival rates, respectively; to study the effect of IFN on patients with intermediate and advanced HCC, the primary and secondary outcome measures were tumor response and survival rate, respectively. No public year restriction was used in this study; therefore, both the World Health Organization criteria and the Response Evaluation Criteria in Solid Tumors were considered for the assessment of tumor response [22]–[24]. Studies were excluded, if publication was just abstract or we could not get a copy of fully published manuscrip. Studies that aimed to evaluate the effect of IFN on HCC prevention were also excluded.

### Assessment of methodological quality

The methodological quality of each RCT was assessed according to the Cochrane Collaboration's tool for assessing risk of bias [25].

### Data collection

Independent data extraction was conducted by two reviewers. The extracted data include the following: publication data (the first author's name, year of publication, and country of population studied), study design, sample size, patient characteristics (mean age and sex ratio), tumor characteristics, therapy protocols, outcome measures, and method of measurement. In studies involving RCT with multiple groups, only the experimental and control groups associated with this study were extracted. If data were unavailable, authors were contacted via e-mail for additional information.

### Level of evidence

The level of evidence of outcomes was assessed using the Grades of Recommendation, Assessment, Development and Evaluation (GRADE) approach [26]. In addition, the GRADE profiler3.6 software was used to create the evidence profile [27].

### Data Analysis

Data were processed in accordance with the Cochrane Handbook. The time to first recurrence and the overall survival were calculated from the date of randomization to the date of diagnosis of recurrence and death (or to the date of the last follow up). The intention-to-treat method was used. Intervention effects were expressed with relative risks (RRs) and associated 95% confidence intervals (CIs) for dichotomous data but with mean differences and 95% CIs for continuous data. Heterogeneity among studies was estimated using the Chi-square test and I2 test (P>0.05 and I2<50% indicate acceptable heterogeneity between the pooled studies) [28]. The fixed-effects model was first used for meta-analysis; if heterogeneity was present, the random-effects model was used.

Subgroup analysis was used to examine the influence of various exclusion criteria on the overall risk estimate. All statistical analyses were performed using RevMan (version 5.1.0; The Cochrane Collaboration). If considerable variation was noted among studies, a brief qualitative analysis of evidence was presented.

## Results

### Search results and study characteristics

Our search yielded 288 potentially relevant studies. among which, thirteen studies were selected for the present meta-analysis after reading the abstracts and full texts. Three articles [29]–[31] reported the same RCT. We included the article with the most complete information. Figure S1 shows the process of literature search and study selection.

Thirteen RCTs [30], [32]–[43] (n = 1344, 675 treated with IFN) were eligible for this study. Eight studies [30], [34], [37]–[41], [43] aimed to investigate the effect of adjuvant IFN on HCC patients after curative treatment with surgical resection or local ablation therapy. Five [32], [33], [35], [36], [42] studies evaluated the effect of IFN on intermediate and advanced HCC patients. Eleven RCTs studied in Asian populations. Most of the patients infected with viral hepatitis. All studies reported that the basic parameters between the two groups were not statistically different. Table S1 shows the characteristics of the studies. The regimen of IFN is tabulated in Table S2.

### Results of methodological quality assessment and the level of evidence

The judgments about each risk of bias item for each included study are shown in Figure S2. Although all studies described randomization, four RCTs did not adequately describe methods of random sequence generation [32]–[34], [37]. Eight studies did not adequately describe allocation concealment [30], [32]–[34], . Only one study kept patients blind by using oral vitamin B complex as placebo; however, it was not an appropriate placebo of IFN. The outcome measurements of this study were objective, and not likely to be influenced by the lack of blinding. Hence, we considered detection bias as low risk. In one study[41], adverse effects resulted in discontinuation of high dose IFN treatment in all patients. In all studies, analysis of recurrence rate and survival rate was according to the intention-to-treat principle. In four studies, modified intention-to-treat analysis was used to evaluate tumor response [32], [33], [35], [36]. One study did not reach the expected sample size [35]. Methods of statistical analysis were well described in all but four studies [32], [34], [36], [37]. Table S3 summarizes the quality of evidence for each outcome measurement.

### Effect of IFN on HCC patients after curative therapy

Considering the overall pool of patients, regardless of the etiology and the approach they received as curative thrapy. IFN was associated with a lower recurrence rate at each year. The combined RR (95% CI) and P value for the 1-, 2-, 3-, 4-, and 5-year recurrence rates were 0.85 (95% CI, 0.73–0.99; P = 0.04), 0.76 (95% CI, 0.6–0.96; P = 0.02), 0.82 (95% CI, 0.7–0.96; P = 0.01), 0.79 (95% CI, 0.68–0.91; P = 0.0009), and 0.83 (95% CI, 0.74–0.93; P = 0.002), respectively (Figure S3). The quality of evidence at each year was low for the 1-, 2-, 3-, and 4-year recurrence rates but moderate for the 5-year recurrence rate. Only one study [41] showed that IFN was associated with better overall survival in HCC patients after curative therapy and the study size was small, 40 patients in each group. Therefore, we didn't perform the meta-analysis.

Exploratory subgroup analysis after stratification by the type of viral hepatitis showed that IFN reduced the 2-, 3-, 4-, and 5-year recurrence rates of HCC patients infected with HCV. Administration of IFN did not affect the 1-year recurrence rate (Figure S4). All studies reported that IFN could not improve the overall survival of HCV-realated HCC patients after curative therapies. The effect of IFN on patients with HBV-related HCC could not be determined because of the small sample size. Subset analysis on patients who received IFN after surgical resection showed that IFN reduced the 4-and 5-year recurrence rates (Figure S5). Because only one study of Lo CM showed that IFN could improve the survival, the meta-analysis of survival rate in HCC patients after surgical resection was not done.

Among the HCC patients infected with HCV, sustained virologic response(SVR) and biochemical response (BR) reported in each study ranged from 0% to 28.6% and 7% to 42.9%, respectively. SVR and BR were not assessed in studies that recruited patients with HBV infection.

### Effect of IFN on intermediate and advanced HCC patients

Meta-analysis was not appropriate in the analysis of IFN for intermediate and advanced HCC patients. Two studies recruited nonpreviously treated HCC patients, and the control group received no antitumor therapy [33], [35]. However, their results were significantly different. Lai et al. [33] reported that IFN-treated patients have significantly better survival and tumor response than control patients. Llovet et al. [35] found no significant difference in survival and tumor response between the two groups. One study compared the effect of IFN with that of doxorubicin [32]. In this study, two IFN groups had different doses (i.e.,18×106 IU/m2 daily and 50×106 IU/m2 thrice weekly). Finally, no significant differences in survival, tumor shrinkage, or side-effects were found between the two regimes of IFN. They were grouped together and associated with better survival and more patients with tumor regression as well as less patients with tumor progression. One study evaluated the efficacy of combined therapy with intra-arterial cisplatin infusion and systemic a dministration of IFN-α as a palliative treatment for patients with major portal vein thrombosis or distant metastasis [36]. The results suggested that combined therapy with intra-arterial cisplatin infusion and systemic IFN-α is associated with better survival and partial response. Compared with transarterial chemoembolization, combined therapy with systemic IFN treatment reduces recurrence and improves the survival of patients with HBV-related HCC [42].

### Safety of IFN

All included studies reported that the most common adverse effects of IFN treatment are flu-like symptoms (fever, fatigue, and myalgia) and myelosuppression (leucocytopenia and thrombocytopenia), which occurred d in almost every patient. Less common adverse effects include neuropsychiatric consequences (depression, dementia,and mental confusion), hepatotoxicity, and development or exacerbation of autoimmune disease, particularly thyroiditis. IFN was well tolerated in all studies but one [41], which reported that all patients administerd with high-dose IFN discontinued the treatment because of adverse effects. Table S2 shows the dose modification rate and treatment discontinuation rate in each study.

## Discussion

We analyzed the effect of IFN on HCC patients who underwent curative therapy. The results showed that IFN was associated with lower recurrence rate. Patients with HBV-related HCC and HCV-related HCC are definitely different subgroups, which might be associated with different responses to IFN. We performed subgroup analysis to evaluate the effect of IFN on the different subsets of population stratified by the type of viral hepatitis. IFN reduced the 2-, 3-, 4-, and 5-year recurrence rates of HCV-related HCC patients, but it did't improve the survival of this population subset. We could not assess the effect of IFN on HBV-related HCC patients because of the small sample size. Curative therapy contains different types of treatment options, among which surgical resection is the most effective. The outcomes of patients who underwent surgical resection and ablation were different with regard to the tumor stage. Therefore, we assessed the effect of IFN on patients who underwent surgical resection. After surgical resection, adjuvant IFN therapy reduced the 4- and 5-year recurrence rates. Almost every study showed that IFN could not improve the overall survival of HCC patients after curative therapy and subgroup of HCC patients after surgical resection. Thus, meta-analysis was considered as unnecessary. In the treatment of intermediate and advanced HCC, the conclusion of each included study remains controversial, and meta-analysis was not possible because of the considerable variation.

The persistent viremia and the presence of underlying cirrhosis are the major factors causing high recurrence rate after curative therapy. Remission of active hepatitis and improvement of hepatic fibrosis explain why IFN could prevent HCC recurrence. Another explanation is the antitumor effect of IFN. Dunk et al. [44] reported that IFN exerts potent growth inhibitory effects on PLC/PRF/5 both in vitro and in vivo. Wang et al. [45] concluded that IFN-α could inhibit metastasis and recurrence of human HCC after curative resection in nude mice. The antitumor effect of IFN can also be supported by the results of two RCTs [32], [36], which showed that high-dose IFN exerts better antitumor effect than doxorubicin. Furthermore, IFN was effective in the reduction of extrahepatic tumor burden. The following mechanisms contribute to the antitumor effect of IFN. First, IFN can cause the induction of pro-apoptotic genes. Takaoka et al. showed that IFN-α/β induces the transcription of the p53 gene, accompanied by an increase in p53 protein level [46]. Second, IFN has direct effects on malignant cells [44], [47]. Third, IFN can inhibit angiogenesis [45]. Last, it can augment antitumor immune responses [48]. Recurrence prevention is generally accepted to favor better survival, and the use of IFN can improve liver function and indicate further treatment of HCC. However, almost every study showed that IFN could not improve the overall survival. It may be attributed to the effects of critical factors, including recurrence pattern and further treatment for recurrence, which are associated with the overall survival of patients.

IFN α was widely used in the included studies, except in the study of Ikeda [34]. The subtypes of IFN α, including IFN α-1b, 2a, and 2b were used in some studies. The dose varied from 3×l0^6^ IU to 50×l0^6^ IU/m^2^. IFN has a dose-dependent effect and so is the side effect. Three studies set two groups, in which patients received different IFN regimens [32], [37], [41]. Two studies reported that no difference in outcome measures and side effects was present between the two IFN groups 32,37. In the study of Lo, IFN was poorly tolerated by postoperative patients at a dose of 30×l0^6^ IU/m^2^ thrice weekly [41]. By contrast, IFN was well tolerated in most cases even at a dose of 50×10^6^ IU/m^2^ thrice weekly in the two studies performed by Lai[32], [33], who recruited inoperable HCC patients. The reason for the difference in IFN tolerance among patients is unclear. Some scholars assumed that different ethnic origins could justify the variation.

Many issues remained in the use of IFN on HCC patients. First, a better regimen is still needed. Further studies explore the effect of IFN combined with other interventions should be performed, such as combination of IFN and ribavirin, combination of cisplatin, interferon, doxorubicin and fluorouracil [49]. Second, the different responses of patient to IFN should be determined to identify the best treatment course for each patient. For instance, whether HCC patients with different HCV genotypes respond differently to IFN should be confirmed. Third, whether IFN is accompanied by other effects should be investigated. Zhuang et al. [50] reported that IFN-α treatment significantly suppresses tumor growth of HCC but relatively increases the number of circulating tumor cells. This result might be attributed to the enhanced tumor hypoxia as well as the up-regulation of metastasis-related genes, such as HIF-1α, c-met, u-PA, PDGF-A, and IL-8.

Our study may contain potential biases. First, placebo control is impractical, which might lead to performance bias. Second, the regimen of IFN varied widely among studies. The interval between previous therapies and IFN administration was not addressed in most of the studies, which might be the potential factor that affects the efficacy. Third, most of the studies involve HCC patients infected with HCV. The number of studies on HCC patients infected with HBV is limited. No data on IFN role in alcohol or NAFLD related HCC. Fourth, subgroup analysis comparing the effect of IFN in different subsets of population may not be appropriate because of the small sample size of each subgroup. Fifth, few studies when examining extended followup times. Finally, the level of evidence for the 1-, 2-, 3-, and 4-year recurrence rates is low.

## Conclusion

In summary, after curative therapies, adjuvant IFN reduced the 1-, 2-, 3-, 4-, and 5-year recurrence rates of HCC patients (regardless of the etiology). In the subgroup of HCV-related HCC patients, IFN reduced the 2-, 3-, 4-, and 5-year recurrence rates after curative therapies. Adjuvant IFN therapy merely reduced the 4- and 5- recurrence rates of HCC patients who received surgical resection as curative therapy. IFN did not improve the survival of HCV-related HCC patients after curative therapy. Whether IFN is effective for intermediate and advanced HCC patients could not be determined because of insufficient data. The toxicity of IFN was acceptable.

## Supporting Information

Figure S1 **Flow chart of literature search and study selection.**
(TIF)

Figure S2 **Risk of bias summary: judgements about each risk of bias item for each included study.**
(TIF)

Figure S3 **Forest plots describing the meta-analysis of recurrence rate at each year among the overall pool of patients.** (curative therapies + IFN vs. curative therapies).
(TIF)

Figure S4 **Forest plots describing the meta-analysis of recurrence rate at each year among the subgroup of HCV-related HCC patients.** (curative therapies + IFN vs. curative therapies).
(TIF)

Figure S5 **Forest plots describing the meta-analysis of recurrence rate at each year among the subgroup of resected HCC patients.** (surgical resection + IFN vs. surgical resection).
(TIF)

Table S1 **Characteristics of included studies.**
(DOC)

Table S2 **IFN regimen.**
(DOC)

Table S3 **Evidence profile for IFN in the treatment of HCC.**
(DOC)

File S1 **PRISMA checklist.**
(DOC)
