# Supplementary figures and images for: Effect and Safety of Interferon for Hepatocellular Carcinoma: A Systematic Review and Meta-Analysis
Source: PLoS One. 2013 Sep 17;8(9):e61361. doi: 10.1371/journal.pone.0061361 (PMC3775819; doi:10.1371/journal.pone.0061361)

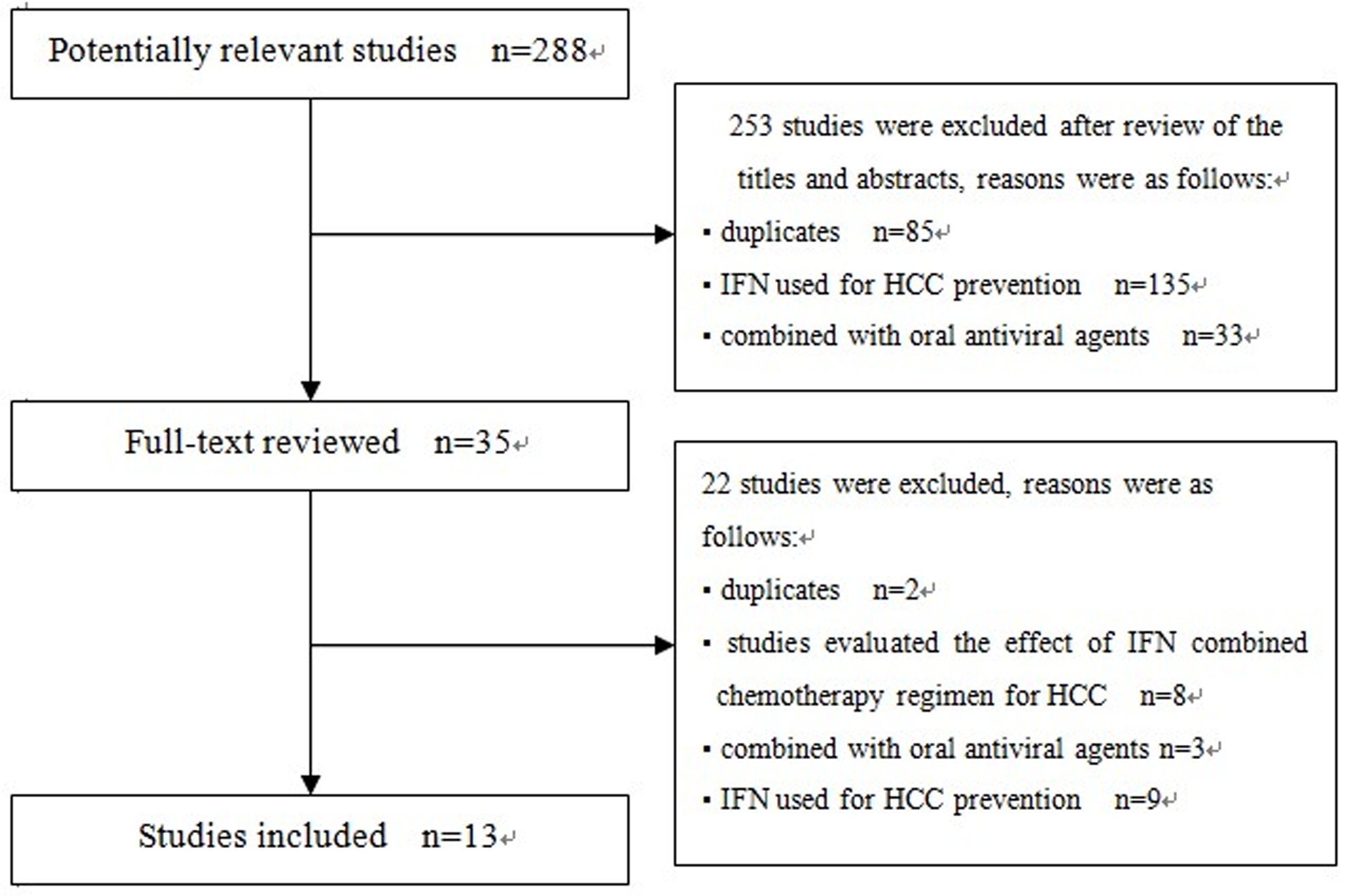

Supplement: Figure S1 — Flow chart of literature search and study selection. (TIF) [file pone.0061361.s001.tif]

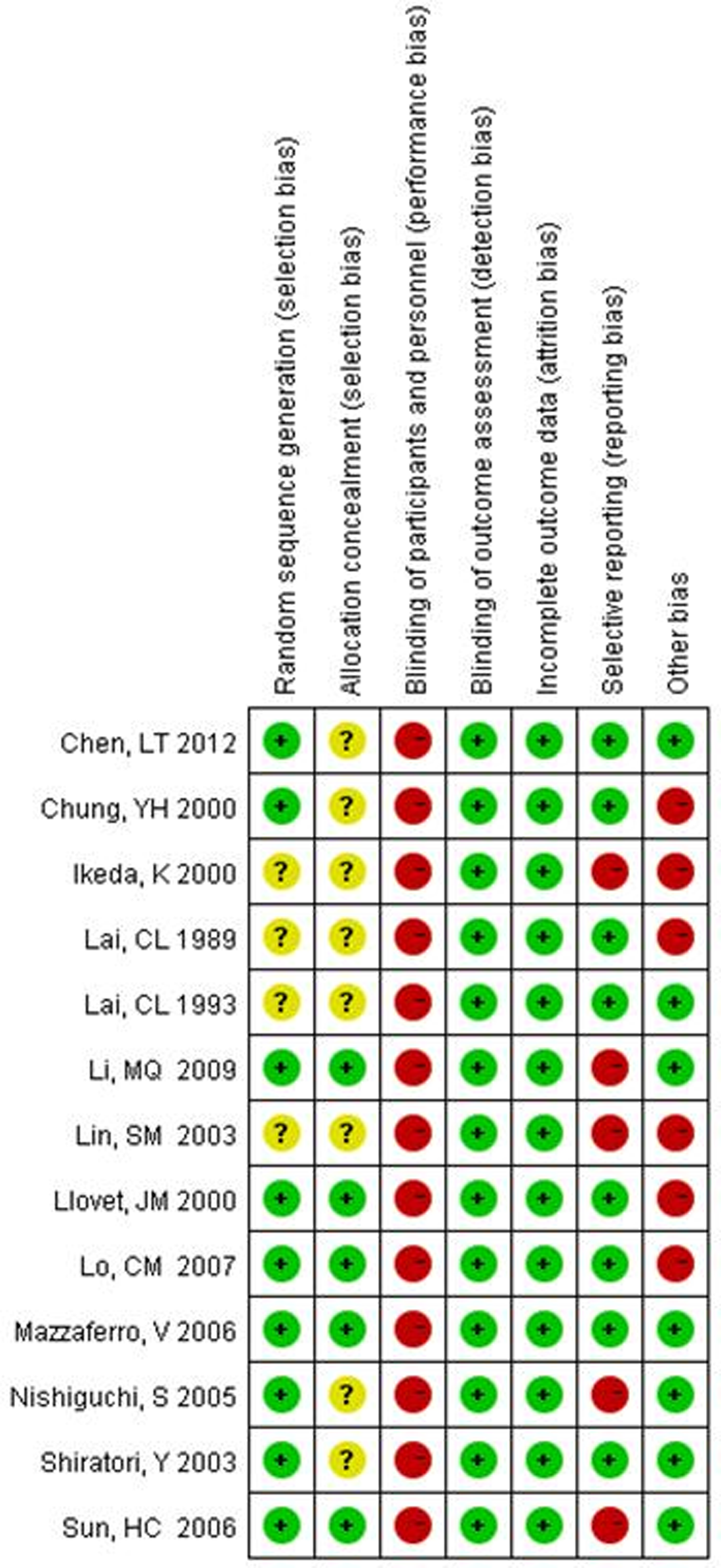

Supplement: Figure S2 — Risk of bias summary: judgements about each risk of bias item for each included study. (TIF) [file pone.0061361.s002.tif]

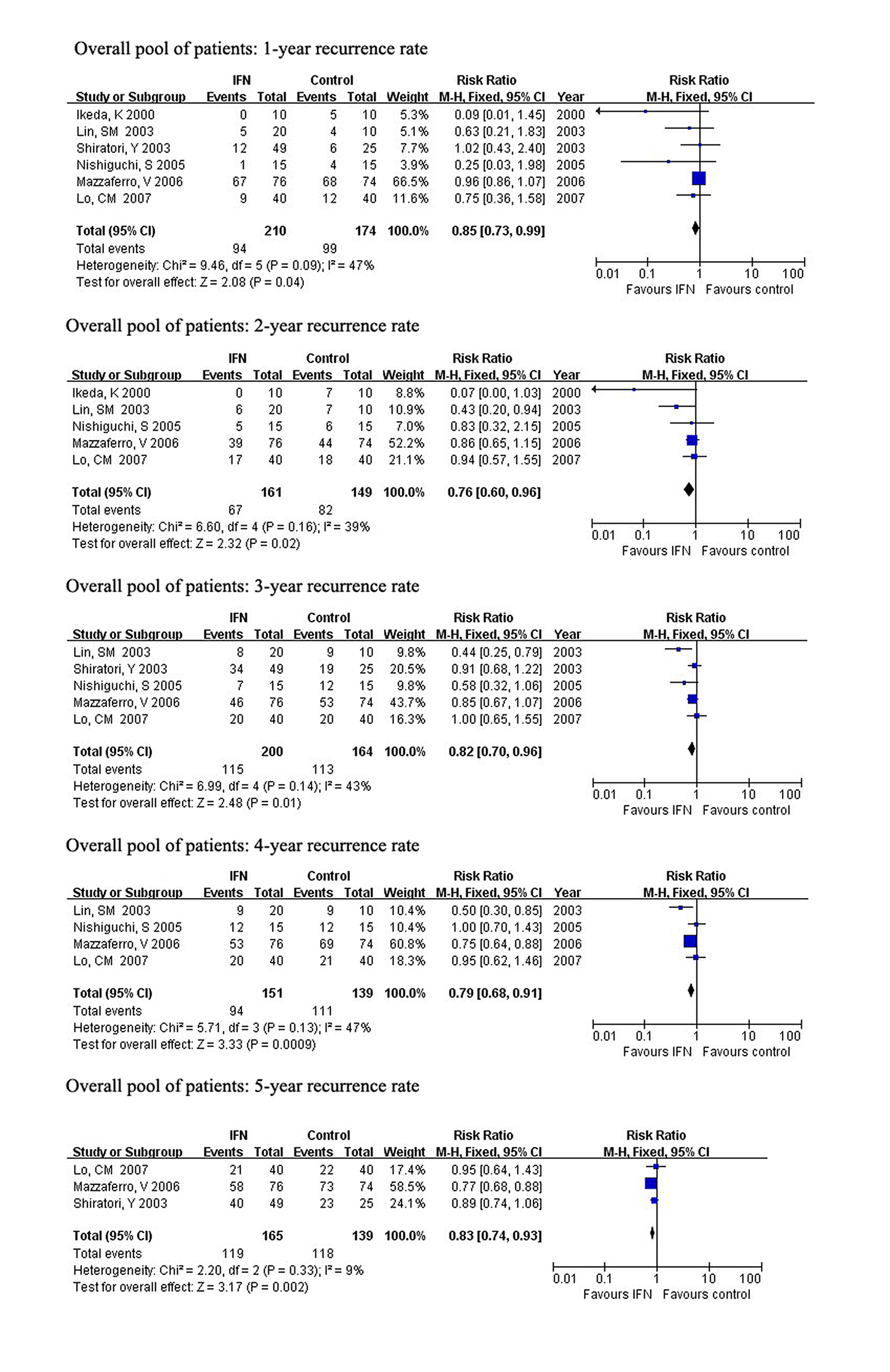

Supplement: Figure S3 — Forest plots describing the meta-analysis of recurrence rate at each year among the overall pool of patients. (curative therapies + IFN vs. curative therapies). (TIF) [file pone.0061361.s003.tif]

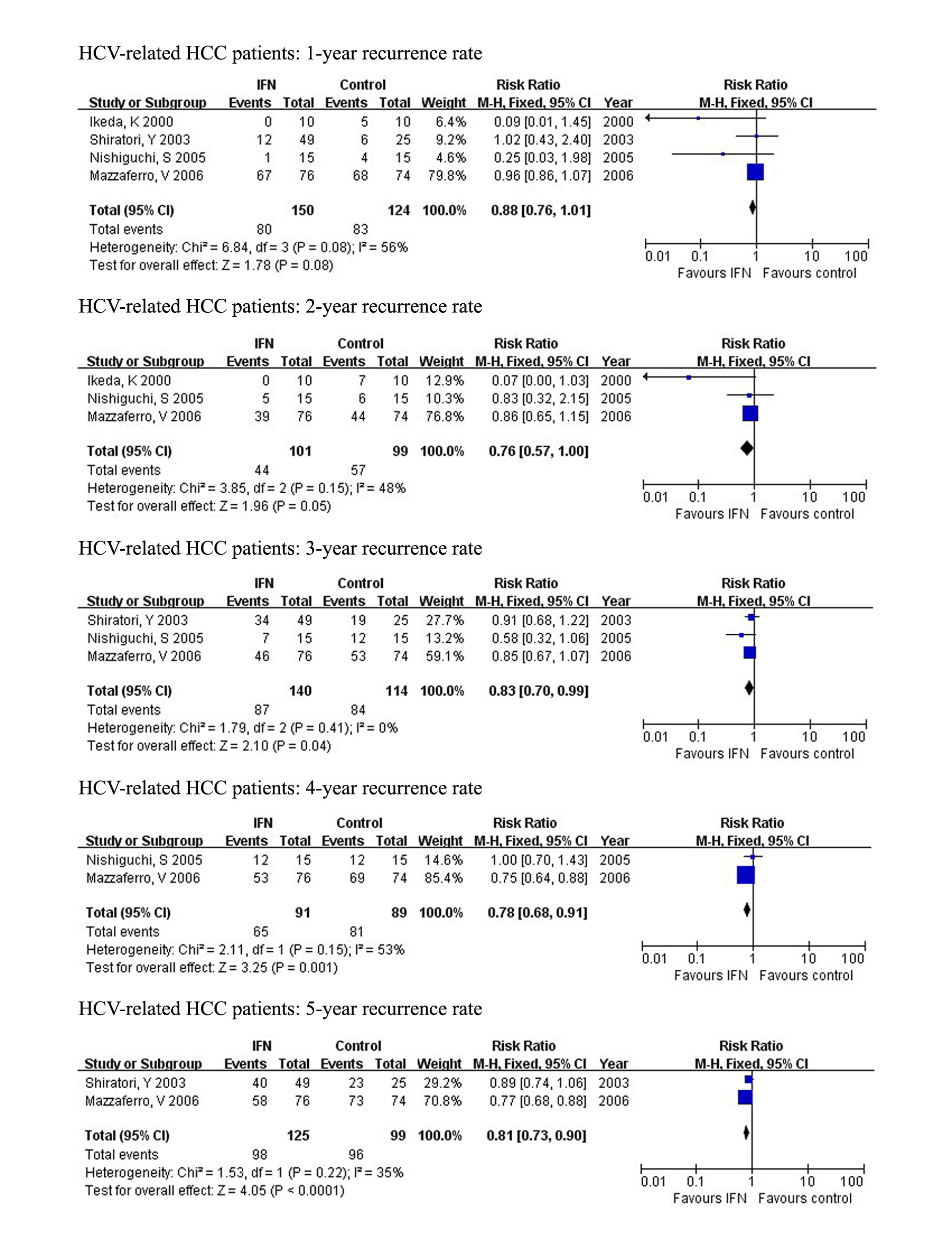

Supplement: Figure S4 — Forest plots describing the meta-analysis of recurrence rate at each year among the subgroup of HCV-related HCC patients. (curative therapies + IFN vs. curative therapies). (TIF) [file pone.0061361.s004.tif]

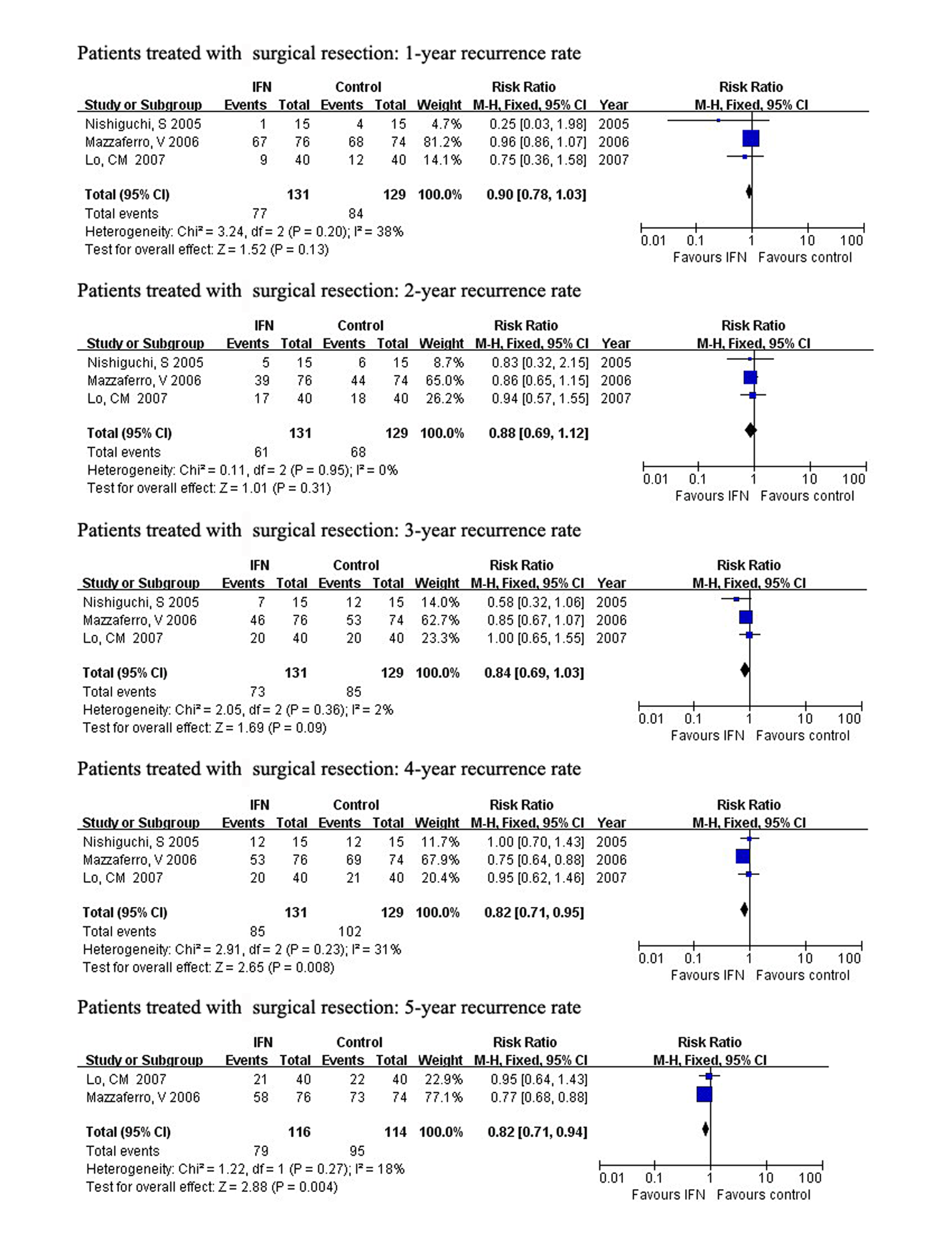

Supplement: Figure S5 — Forest plots describing the meta-analysis of recurrence rate at each year among the subgroup of resected HCC patients. (surgical resection + IFN vs. surgical resection). (TIF) [file pone.0061361.s005.tif]
