# Supplementary material for: Effect and Safety of Interferon for Hepatocellular Carcinoma: A Systematic Review and Meta-Analysis
Source: PLoS One. 2013 Sep 17;8(9):e61361. doi: 10.1371/journal.pone.0061361 (PMC3775819; doi:10.1371/journal.pone.0061361)
Supplement: Table S1 — Characteristics of included studies. (DOC) [file pone.0061361.s006.doc]

**Table S1 Characteristics of included studies**

| Author/Ref. | Year / country | Previous therapy | Group | No. | Median age | Gender (M:F) | Etiology (%) | | | Interventions | Median follow-up time (m) |
| --- | --- | --- | --- | --- | --- | --- | --- | --- | --- | --- | --- |
|  |  |  |  |  |  |  | HBV | HCV | Others |  |  |
| Lai, CL ^[31]^ | 1989 China | Never | IFN | 22 | NA | NA | 88 | NA | NA | IFN | NA |
|  |  |  | Control | 47 | NA | NA | 88 | NA | NA | ADM | NA |
| Lai, CL ^[32]^ | 1993 China | Never | IFN | 35 | 59 | 32:3 | 94.3 | NA | NA | IFN | NA |
|  |  |  | Control | 36 | 60 | 29:7 | 94.4 | NA | NA | vitamin B | NA |
| Ikeda, K ^[33]^ | 2000 Japan | Resection or PEI | IFN | 10 | 60 | 7:3 | 0 | 100 | 0 | IFN | 25 (2-34.6) |
|  |  |  | Control | 10 | 64.5 | 6:4 | 0 | 100 | 0 | ST. |  |
| Llovet, JM ^[34]^ | 2000 Spain | Never | IFN | 30 | 61 | 27:3 | 6.7 | 76.7 | 16.7 | IFN | 18±22 |
|  |  |  | Control | 28 | 63 | 22:61 | 0 | 78.6 | 21.4 | ST. | 12±55 |
| Chung YH ^[35]^ | 2000 Korea | Never | IFN | 19 | 49 | NA | NA | NA | NA | TAE+IFN | NA |
|  |  |  | Control | 23 | 52 | NA | NA | NA | NA | TAE | NA |
| Lin SM ^[36]^ | 2003 China | PAI | IFN | 20 | 61.5 | 7:3 | 45 | 55 | 0 | IFN | 27 (4-53) |
|  |  |  | Control | 10 | 59 | 16:4 | 60 | 40 | 0 | ST. |  |
| Shiratori Y ^[37]^ | 2003 Japan | PEI | IFN | 49 | 61 | 35:14 | 0 | 100 | 0 | IFN | 85.2±15.6 |
|  |  |  | Control | 25 | 63 | 17:8 | 0 | 100 | 0 | ST. |  |
| Nishiguchi, S^[29]^ | 2005 Japan | Resection | IFN | 15 | 61.9 | 15:0 | 0 | 100 | 0 | IFN | 60.6 (52.6,66.9) |
|  |  |  | Control | 15 | 60 | 15:0 | 0 | 100 | 0 | ST. | 49.6 (39.8,68.5) |
| Mazzaferro,V^[38]^ | 2006 Italy | Resection | IFN | 76 | 65 | 61:15 | 0 | 100 | 0 | IFN | 45 |
|  |  |  | Control | 74 | 67 | 51:23 | 0 | 100 | 0 | ST. |  |
| Sun, HC^[39]^ | 2006 China | Resection | IFN | 118 | 52.2 | 106:12 | 100 | 0 | 0 | IFN | 36.5 |
|  |  |  | Control | 118 | 50.4 | 102:16 | 100 | 0 | 0 | ST. |  |
| Lo, CM ^[40]^ | 2007 China | Resection | IFN | 40 | 49 | 31:9 | 93 | 3 | 4 | IFN | minimum follow-up 30 |
|  |  |  | Control | 40 | 54 | 34:6 | 88 | 5 | 7 | ST. |  |
| Li, MQ ^[41]^ | 2009 China | Never | IFN | 108 | NA | 77:31 | 100 | 0 | 0 | TACE+IFN | 24.8 |
|  |  |  | Control | 108 | NA | 74:34 | 100 | 0 | 0 | TACE |  |
| Chen, LT ^[42]^ | 2012 China | Resection | IFN | 133 | NA | 108:25 | 79.7 | 20.3 | 0 | IFN | 63.8 (60.8-66.9) |
|  |  |  | Control | 135 | NA | 112:23 | 80 | 19.3 | 0.7 | ST. |  |

PEI, percutaneous ethanol injection; PAI, percutaneous acetic acid injection; TAE, transarterial embolization;

TACE, transarterial chemoembolization; ADM, Doxorubicin; ST., Symptomatic treatment;

NA, Not available
