# Supplementary material for: Effect and Safety of Interferon for Hepatocellular Carcinoma: A Systematic Review and Meta-Analysis
Source: PLoS One. 2013 Sep 17;8(9):e61361. doi: 10.1371/journal.pone.0061361 (PMC3775819; doi:10.1371/journal.pone.0061361)
Supplement: Table S2 — IFN regimen. (DOC) [file pone.0061361.s007.doc]

**Table S**2 IFN regimen

| Author/Ref. | IFN type | Regimen | Dose reduction rate (%) | Discontinuation rate(%) |
| --- | --- | --- | --- | --- |
| Lai, CL ^[31]^ | IFNα | IFN group 1: 18 x 10^6^ IU/m^2^, daily  IFN group 2: 50 x 10^6^ IU/ m^2^, 3 times weekly | 5/47(10.6) | 2/47(4.3) |
| Lai, CL ^[32]^ | IFNα-2a | 50 x l0^6^ IU/m^2^, 3 times weekly | 12/35(34.3) | 0 |
| Ikeda, K ^[33]^ | IFN-β | 6 x l0^6^ IU, twice a week for 36 months | 1/10(10) | 0 |
| Llovet, JM ^[34]^ | IFNα-2b | 3 x 10^6^ IU, 3 times a week for 1 year | NA | 13/30(43.3) |
| Chung YH ^[35]^ | IFNα-2b | 3 x l0^6^ IU/m^2^, 3 times a week | NA | 0 |
| Lin SM ^[36]^ | IFNα-2b | IFN group 1: 3 x 10^6^ IU, 3 times a week for 24 months  IFN group 2: 3 x 10^6^ IU daily for 10 days every month for 6 months followed by3 x 10^6^ IU daily for 10 days every 3 months for an additional 18 months | 10/20(50) | 3/20(15) |
| Shiratori Y ^[37]^ | IFNα | 6 x 10^6^ IU, 3 times weekly for 48 weeks | 6/49(12.2) | 9/49(18.4) |
| Nishiguchi, S^[29]^ | IFNα | 6 x 10^6^ IU daily for 2 weeks, then 3 times weekly for 14 weeks, and finally twice weekly for 88 weeks | NA | 3/15(20) |
| Mazzaferro,V^[38]^ | IFNα-2b | 3 x 10^6^ IU, 3 times every week for 48 weeks | 9/76(11.8) | 6/76(7.9) |
| Sun, HC^[39]^ | IFNα-1b | 3 x 10^6^ IU, twice weekly for 2 weeks, then 5 x 10^6^ IU, 3 times weekly for 18 months | 23/106(21.7) | 5/106(4.7) |
| Lo, CM ^[40]^ | IFNα-2b | IFN group 1: 10 x l0^6^ IU/m^2^, 3 times weekly for 16 weeks  IFN group 2: 30 x l0^6^ IU/m^2^, 3 times weekly for 16 weeks | 22/40(55) | 3/40(7.5) |
| Li, MQ ^[41]^ | IFNα-1b | 3 x 10^6^ IU, 3 times every week for 48 weeks | NA | 1/108(9.3) |
| Chen,LT^[42]^ | IFNα-2b | 5 days per week for 5 weeks with the dosage escalated from 1 x 10^6^ IU to 5 x 10^6^ IU during the first week and held at 5 x 10^6^ IU for the next 4 weeks, and then administered thrice weekly for an additional 48 weeks | 33/133(24.8) | 5/133(3.8) |
