# Supplementary material for: Effect and Safety of Interferon for Hepatocellular Carcinoma: A Systematic Review and Meta-Analysis
Source: PLoS One. 2013 Sep 17;8(9):e61361. doi: 10.1371/journal.pone.0061361 (PMC3775819; doi:10.1371/journal.pone.0061361)
Supplement: Table S3 — Evidence profile for IFN in the treatment of HCC. (DOC) [file pone.0061361.s008.doc]

**Table S3 Evidence Profile for IFN in the treatment of HCC**

|  | **Evidence Profile** | **RR(95% CI)** | **P Value** | **Risk**  **Without**  **Treatment** | **Risk With**  **Treatment**  **(95% CI)** | **Quality of**  **Evidence** |
| --- | --- | --- | --- | --- | --- | --- |
| 1-year recurrence rate | Serious methodological limitations  Inconsistent, direct, precise,  No publication bias detected | 0.85  (0.73- 0.99) | 0.04 | 52(3-94)/1000 population | 85(6-154)/1000 population |  LOW |
| 2-year recurrence rate | Serious methodological limitations  Inconsistent, direct, precise,  No publication bias detected | 0.76  (0.6 - 0.96) | 0.02 | 143(24-238)/1000 population | 132(22-220)/1000 population |  LOW |
| 3-year recurrence rate | Serious methodological limitations  Inconsistent, direct, precise,  No publication bias detected | 0.82  (0.7 - 0.96) | 0.01 | 137(30-228)/1000 population | 124(28-207)/1000 population |  LOW |
| 4-year recurrence rate | Serious methodological limitations  Inconsistent, direct, precise,  No publication bias detected | 0.79  (0.68 - 0.91) | 0.0009 | 178(76-272)/1000 population | 168(72-256)/1000 population |  LOW |
| 5-year recurrence rate | Serious methodological limitations  Consistent, direct, precise,  No publication bias detected | 0.83  (0.74 - 0.93) | 0.002 | 156(64-239)/1000 population | 144(59-221)/1000 population |  MODERATE |
